# One Health Approach to Globalizing, Accelerating, and Focusing Amphibian and Reptile Disease Research—Reflections and Opinions from the First Global Amphibian and Reptile Disease Conference

**DOI:** 10.3201/eid2910.221899

**Published:** 2023-10

**Authors:** Matthew J. Gray, Robert J. Ossiboff, Lee Berger, Molly C. Bletz, E. Davis Carter, Joseph A. DeMarchi, Leon Grayfer, David Lesbarrères, Daniel A. Malagon, An Martel, Debra L. Miller, Frank Pasmans, Lee F. Skerratt, Anastasia E. Towe, Mark Q. Wilber

**Affiliations:** University of Tennessee, Knoxville, Tennessee, USA (M.J. Gray, E.D. Carter, J.A. DeMarchi, D.L. Miller, A.E. Towe, M.Q. Wilber);; University of Florida, Gainesville, Florida, USA (R.J. Ossiboff);; University of Melbourne, Melbourne, Victoria, Australia (L. Berger, L.F. Skerratt);; University of Massachusetts, Amherst, Massachusetts, USA (M.C. Bletz);; George Washington University, Washington, DC, USA (L. Grayfer);; National Wildlife Research Centre, Ottawa, Ontario, Canada (D. Lesbarrères);; Clemson University, Clemson, South Carolina, USA (D.A. Malagon);; Ghent University, Ghent, Belgium (A. Martel, F. Pasmans)

**Keywords:** One Health, reptiles, amphibians, Global Amphibian and Reptile Disease conference, GARD, herpetology, herpetofauna, herpetofaunal disease

## Abstract

The world’s reptiles and amphibians are experiencing dramatic and ongoing losses in biodiversity, changes that can have substantial effects on ecosystems and human health. In 2022, the first Global Amphibian and Reptile Disease Conference was held, using One Health as a guiding principle. The conference showcased knowledge on numerous reptile and amphibian pathogens from several standpoints, including epidemiology, host immune defenses, wild population effects, and mitigation. The conference also provided field experts the opportunity to discuss and identify the most urgent herpetofaunal disease research directions necessary to address current and future threats to reptile and amphibian biodiversity.

The One Health definition recently developed by the One Health High-Level Expert Panel states: “One Health is an integrated, unifying approach that aims to sustainably balance and optimize the health of people, animals and ecosystems” (*1*). One Health recognizes that the health of humans, domestic and wild animals, plants, and the wider environment (including ecosystems) are closely linked and interdependent. The approach mobilizes multiple sectors, disciplines, and communities at various levels of society to work together to foster wellbeing and tackle threats to health and ecosystems, while addressing the collective need for clean water, air and energy, safe and nutritious food, taking action on climate change, and contributing to sustainable development (*1*). Governments and health organizations have adopted One Health approaches, and such initiatives are now being implemented at universities and in professional societies (*2*). A crux of the One Health approach is the sharing of information across host–pathogen systems to identify parallels and enable the inception and institution of strategies that broadly combat emerging infectious diseases (*3*).

Although the connection of herpetofauna (reptiles and amphibians) to One Health may not be immediately clear, those vertebrate animals are essential components of ecosystems and are experiencing broad and unprecedented losses in biodiversity (*4*,*5*). Moreover, the decline and loss of reptile and amphibian species can have direct effects on human health (*6*,*7*). Many reptiles and amphibians are insectivores and consume various insect pests that can have negative effects on agriculture and human health. For example, cases of malaria increased in many regions of Central America in the years directly after asynchronous amphibian declines (*7*). Many herpetofaunal species also have human biomedical value. For example, amphibians are a model for organogenesis research because many can regenerate their limbs and for bioprospecting because they produce chemicals that have antimicrobial effects (e.g., inactivating HIV) and strong analgesic properties (*8*–*12*). Because of their biphasic life cycle, amphibians also are considered excellent bioindicators for terrestrial and aquatic ecosystem health at a time when the degradation of both habitats impedes quality of human life. Further, the biomass of amphibians and reptiles in certain areas can exceed that of all other vertebrate animals, which means they play a major role in nutrient cycling (functioning as prey and predators) and sequester large quantities of carbon, thereby buffering global climate change (*13*–*16*). Reptiles and amphibians are consumed as food in certain societies, and some serve as pets, contributing positively to public physical and mental health (*17*).

In August 2022, the first Global Amphibian and Reptile Disease (GARD) Conference was organized in Knoxville, Tennessee, USA, using One Health approaches as a guiding principle. More than 250 participants representing 25 countries on 6 continents participated in the hybrid conference, which included in-person activities and an online platform for virtual participants (*18*). The program consisted of 8 keynote addresses, 5 focal talks, 105 oral research presentations, and 31 poster presentations. Students delivered 52% of oral presentations and 70% of poster presentations. Diversity, equity, and inclusion were enhanced by providing 40 travel grants to students and early career professionals from 9 countries and 13 US states (83% awarded to minorities and women). In addition, 5 professional development workshops provided opportunities to expand disease diagnostic, analytical, and research skills. 

The program was divided into 8 topic-focused sessions, each ending with a moderated panel discussion to identify information gaps and urgent research needs on herpetofaunal diseases. Diseases receiving the greatest attention during the conference included amphibian chytridiomycosis (56%), amphibian and reptile ranavirosis (11%), and snake ophidiomycosis (8%). However, other unique or emerging diseases, including amphibian perkinsiosis, reptile invasive pentastomiasis caused by *Raillietiella orientalis*, snake serpentoviruses, and snake *Parananizziopsis*, were also highlighted. Despite the various pathogens discussed, other important reptile and amphibian pathogens, including *Mycoplasma* spp., *Chlamydia* spp., herpesviruses, reptarenaviruses, adenoviruses, ferlaviruses, and testudines intranuclear coccidia, were not addressed. Noninfectious diseases also were not included in presentations but are increasing in importance because habitats and environments are affected by anthropogenic changes, such as pollution. Of note, more abstracts were submitted and presentations delivered on amphibian pathogens (84%) than on reptile pathogens (16%), which might reflect current funding biases.

We identified 4 primary disease categories covered during 2022 GARD Conference: epidemiology, host immune defenses, wild population effects, and mitigation. We summarize the most urgent research directions that were discussed in these areas and offer some considerations for future wildlife health policy and funding.

## Information Gaps and Urgent Research Directions

### Epidemiology

The epidemiology of most reptile and amphibian diseases remains poorly understood. During the conference, participants emphasized the need to rigorously study pathogen spread across countries and ecological barriers to prevent further spread. Indirect transmission pathways, spread of pathogens through fomites, introduction of novel pathogens into naive environments, and sustained transmission events even as populations decline were seen as important areas for study. For example, presentations highlighted that several herpetofaunal pathogens have environmental reservoirs and can exist outside the host for days to months. In addition, some pathogens, such as chytrid fungi and iridoviruses, can persist on or in nonherpetofaunal hosts, such as invertebrates and fish. Carcasses infected with *Batrachochytrium salamandrivorans* and ranaviruses can contribute substantially to postmortem transmission to susceptible hosts. In light of those findings, the importance of implementing good practices to prevent the further spread of pathogens into naive populations was regarded as a high priority. Bridging that knowledge gap will require collaboration among experimentalists, field biologists, diagnosticians, and modelers to combine laboratory and mesocosm results, longitudinal surveillance data, and epidemiologic models, embodying the tenets of a One Health approach.

Because pathogen growth, survival, and transmission often are affected by the external environment, shifting environmental conditions caused by climate change and other factors must be considered when predicting future herpetofaunal health (*19*). Temperature is a critical environmental variable that affects host–pathogen interactions for many disease systems, including herpetofaunal pathogens (*20*,*21*). Evidence suggests that thermal mismatches between a host’s thermal optimum and temperatures experienced may increase disease susceptibility, disease severity, and risk for population decline (*22*). However, presentations and discussions recognized that a major remaining challenge is how to link infection responses of individual hosts measured at varying temperatures in the laboratory to population- and community-level disease responses in the field. A combination of targeted experiments and further development of the metabolic theory of thermal mismatches are needed to overcome this knowledge limitation, further emphasizing the importance of embracing a One Health approach to understanding herpetofaunal diseases in the face of a changing climate.

Historically, studies of herpetofaunal diseases have principally focused on the effect of a single pathogen, largely ignoring the potential for hosts to be co-infected with multiple pathogens and how that might alter disease outcomes. Across multiple disease systems, the conference highlighted the need for more detailed and comprehensive investigations into potential interactions of herpetofaunal pathogens, especially for those that have recently spread to new areas and hosts because of globalization and insufficient prevention of disease spread. For example, some research suggests that ranavirus infection potentiates chytridiomycosis in frogs (*23*), whereas co-infection with ranavirus and *B. dendrobatidis* appears to have limited or no effect in some salamander species (*24*). In parallel, a need exists to consider communities of hosts that may be infected by one or more pathogens if we are to better understand the effect of these diseases on the ecosystem (*25*,*26*). Because the outcome of pathogen co-infection probably is dependent on complex interactions among the hosts, pathogens, and environment (*27*), multidisciplinary collaborations using a One Health approach are most likely to lead to accurate predictions of co-infection outcomes under novel conditions.

### Host Immune Defenses

The nature and magnitude of host immune responses play prominent roles in defining infectious disease outcomes (*28*). Although immune responses to various pathogens are well characterized in mammal hosts, much less is known about general and pathogen-specific immune responses of reptiles and amphibians (*29*,*30*). An overarching theme that emerged during the GARD Conference was that herpetofaunal diseases often are multifaceted. Various complex factors, particularly environmental conditions, can serve as major determinants of disease in reptiles and amphibians, and hence, the immune responses to diseases should be investigated with these factors in mind. A need exists to define the extent to which amphibian and reptile fungal infection outcomes depend on successful pathogen clearance versus the capacities of infected animals to minimize inflammation-associated tissue damage while remaining infected. In other words, understanding the factors that influence balances between resistance and tolerance mechanisms is critical. Species- and individual-specific susceptibility differences in immune responses probably are a result of complex interactions among interconnected factors, such as genetics, exposure history to pathogens, environment, metabolism, microbiomes, life stage, and co-infections. Research exploring those interactions will help lead to identifying effective treatments and prophylactic therapies for herpetofaunal diseases. Moreover, we need to determine if such interconnected factors can be manipulated to increase the success of animal repatriation in areas where species have been extirpated by disease. This information will particularly improve reintroduction success of threatened species that are being maintained in ex situ conservation programs.

### Wild Population Effects and Surveillance

Although the field has made great strides toward improving herpetofaunal pathogen identification and characterization, the conference highlighted the generalized need for studies to determine the effects of these pathogens on wild reptile and amphibian populations. Even though the population-level effects of chytridiomycosis are generally well-studied (*31*,*32*), little is known regarding the effects of other herpetofaunal diseases, such as ophidiomycosis and ranavirosis. Unfortunately, longitudinal surveillance studies of wild reptile and amphibian populations are rare (*33*) and consist largely of targeted pathogen screening in presumed stable populations, easily accessed areas (*34*,*35*), or involve reactionary screening after mortality events (*36*,*37*). To better evaluate the health of herpetofaunal populations, we need long-term longitudinal and broad-scale pathogen surveillance studies that use advanced techniques, such as metabarcoding and environmental DNA, ideally linked to population dynamic studies (*38*). Data generated could be used in risk analyses to rank pathogen threats to herpetofauna and help in developing targeted wildlife disease management.

### Mitigation and Policy

Disease management emerged as another theme of the conference. Both curative and prophylactic strategies were proposed that targeted all 3 components of the disease triangle: the host by modulating host defenses, the environment by manipulating conditions to reduce suitability for the pathogen, and the pathogen itself. Most of the work presented at the GARD Conference focused on host-centric strategies, including improving host defenses by using skin probiotic bioaugmentation and vaccination, targeted genetic intervention, and population density reduction. Other presented research centered on manipulating host environments, such as increasing habitat complexity, providing thermal refugia, augmenting micropredators, and applying novel antifungal agents (*39*,*40*). Participants also acknowledged that continued research is needed on potential nontarget consequences of disease management strategies. Of note, recognizing that there probably is no one-size-fits-all approach, integrating multiple strategies may be key to effective disease management (*41*). Overall, although managing diseases in wild herpetofaunal populations probably is feasible, it will require effective collaborations among wildlife managers and scientists, with substantial financial resources and government support to enable an effective response.

One last point repeated throughout the conference was the role that the trade of amphibians and reptiles plays in the global emergence of herpetofaunal diseases. The rapid movement of amphibians and reptiles around the globe through wildlife trade, without adequate measures to ensure animals are pathogen-free, can result in the translocation of pathogens. Sourcing wild herpetofauna for the pet trade carries a continuous risk for introducing novel pathogens to naive regions. Such pathogens have the potential to affect not only animal colonies in captivity but also wild populations of herpetofauna, where they can reduce biodiversity. Although some exceptions exist, including salamander shipments in Europe, animal health certificates generally are not required for internationally traded herpetofauna (*41*). 

During the conference, potential options to mitigate the spread of reptile and amphibian pathogens through trade were discussed. Scientists and several industry representatives discussed creating a healthy trade certification program in the United States for pet amphibians. Other suggestions included creating government programs that could help subsidize appropriate practices to prevent pathogen spread and pathogen testing in wildlife trade. Another suggestion was that, by supporting and expanding healthy captive reptile and amphibian breeding programs, both the international trade of wild herpetofauna and pathogen spread and spillover to new hosts could be reduced. Ultimately, the prevailing theme underscored by the conference was the need for greater industry and government partnerships to work toward healthy (clean) trade and a preference for improved prevention of pathogen spread and testing over regulations that ban the trade of herpetofauna (*42*–*45*).

## A Call for a Funded One Health Approach

The need for government support of wildlife disease monitoring and management programs is a resounding theme within the international research community (*46*–*48*). Adopting comprehensive wildlife health bills that include amphibian and reptile diseases, with a focus on prevention (e.g., clean trade and biosecurity), detection (e.g., early warning systems), and mitigation (preparing action plans that include decision trees and freeing up necessary resources) using a multidisciplinary approach (e.g., scientists, managers, decision makers) is necessary to avert further disease-driven biodiversity loss. Wildlife health legislation could use well-established criteria and government-supported programs for monitoring the health of agricultural animals or aquaculture. Although substantial resources are invested across the globe to monitor public, livestock, and environmental health and respond to threats, few resources have been dedicated to wildlife health programs. As we observed with the SARS-CoV-2 pandemic, the health of humans is inextricably linked to the health of wild and domesticated animals. It is an international responsibility to develop health plans that include wildlife and support actions that conserve global biodiversity. One Health is the framework under which common goals and approaches to managing wildlife and human health can be unified. Considering that amphibians and reptiles represent many of the most imperiled vertebrates on Earth (*31*,*49*,*50*), we urge that steps be taken now by government agencies and other organizations to support herpetofaunal research and develop wildlife health programs that dedicate resources to amphibian and reptile conservation.

A postconference survey deemed the first GARD Conference a success. Greater inclusion of reptile diseases and newly discovered pathogens were suggestions for future conferences. The next GARD Conference will be held in association with the 10th World Congress of Herpetology (https://2024wch10.com) in Malaysia in August 2024 (*51*). Participation in GARD 2024 will be seamless with the World Congress of Herpetology, a single registration fee enabling access to both meetings. Similar to the 2022 meeting, using a One Health approach to studying emerging infectious diseases in herpetofaunal communities will be the guiding principle of the 2024 GARD Conference.
